# Supplementary material for: Different Dynamics of Bacterial and Fungal Communities in Hive-Stored Bee Bread and Their Possible Roles: A Case Study from Two Commercial Honey Bees in China
Source: Microorganisms. 2020 Feb 15;8(2):264. doi: 10.3390/microorganisms8020264 (PMC7074699; doi:10.3390/microorganisms8020264)

Supplementary material Tables

Supplementary material Table1

pH measurements (Significant difference between treatments with One-way ANOVA)

| Sample | PM | BM | PC | BC |
|---|---|---|---|---|
| sample1 | 6.90a | 5.93b | 6.82a | 5.91b |
| sample2 | 6.92a | 5.9b | 6.92a | 5.99b |
| sample3 | 6.89a | 5.88b | 6.99a | 5.88b |
| sample4 | 7.01a | 5.74b | 6.89a | 5.89b |
| sample5 | 6.76a | 5.9b | 7.03a | 5.78b |
| sample6 | 6.79a | 5.82b | 7.01a | 6.02b |
| Average | 6.87±0.03 | 5.86±0.02 | 6.94±0.03 | 5.91±0.03 |

Supplementary material Table2

Colony forming unit/gram (Significant difference between treatments with One-way ANOVA)

| Sample | Fungi | | | | Bacteria | | | |
|---|---|---|---|---|---|---|---|---|
| | PM | BM | PC | BC | PM | BM | PC | BC |
| sample1 | 1200a | 1100a | 2890a | 1300a | 24000a | 6000b | 6800b | 1400c |
| sample2 | 1900a | 1980a | 1000a | 2500a | 11000a | 7800b | 8230b | 720c |
| sample3 | 2100a | 2010a | 1400a | 1600a | 19800a | 8900b | 4680b | 650c |
| sample4 | 1500a | 1760a | 1900a | 2190a | 20100a | 7600b | 4540b | 980c |
| sample5 | 1700a | 1540a | 2560a | 1560a | 14700a | 8000b | 5600b | 1340c |
| sample6 | 1800a | 1300a | 2100a | 1450a | 17300a | 5400b | 5500b | 1100c |
| Average | 1700±129. | 1615±150. | 1975±287. | 1766.6±191. | 17816±186 | 7283.33±538 | 5891.66±572 | 1031.66±126 |

Supplementary material Table3

Number of OTUs

Bacteria OTUs (Significant difference between treatments with t-test)

| Sample | PM | BM | PC | BC |
|---|---|---|---|---|
| sample1 | 325a | 237a | 328a | 196b |
| sample2 | 236a | 238a | 260a | 201b |
| sample3 | 280a | 167a | 153a | 187b |
| sample4 | 271a | 225a | 184a | 132b |
| sample5 | 227a | 232a | 322a | 115b |
| sample6 | 189a | 240a | 347a | 122b |
| Average | 254.67±19 | 223.16±11 | 265.66±33 | 158.83±16 |

Fungi OTUs (Significant difference between treatments with t-test)

| Sample | PM | BM | PC | BC |
|---|---|---|---|---|
| sample1 | 631a | 196a | 343ab | 65b |
| sample2 | 587a | 497a | 392ab | 197b |
| sample3 | 848a | 556a | 318ab | 263b |
| sample4 | 629a | 412a | 693ab | 354b |
| sample5 | 606a | 543a | 396ab | 523b |
| sample6 | 428a | 578a | 483ab | 225b |
| Average | 621.5±54 | 463.66±58 | 437.5±56 | 271.16±63 |

Supplementary material Table 4

Diversity indices

Bacteria

| Simpson | PM | BM | PC | BC |
|---|---|---|---|---|
| Sample1 | 0.95a | 0.76b | 0.86a | 0.81b |
| Sample2 | 0.91a | 0.81b | 0.85a | 0.64b |
| Sample3 | 0.99a | 0.68b | 0.88a | 0.72b |
| Sample4 | 0.94a | 0.47b | 0.95a | 0.66b |
| Sample5 | 0.92a | 0.81b | 0.90a | 0.84b |
| Sample6 | 0.88a | 0.64b | 0.90a | 0.81b |
| Average | 0.93±0.01 | 0.69±0.04 | 0.89±0.01 | 0.75±0.02 |

| Shannon | PM | BM | PC | BC |
|---|---|---|---|---|
| Sample1 | 4.49a | 3.04b | 3.36a | 2.86b |
| Sample2 | 4.02a | 2.90b | 3.24a | 2.00b |
| Sample3 | 4.87a | 2.53b | 2.89a | 2.51b |
| Sample4 | 4.08a | 1.60b | 4.36a | 2.03b |
| Sample5 | 3.99a | 2.86b | 3.46a | 2.99b |
| Sample6 | 3.74a | 2.00b | 3.35a | 2.83b |
| Average | 4.20±0.14 | 2.49±0.19 | 3.44±0.16 | 2.53±0.14 |

| Chao | PM | BM | PC | BC |
|---|---|---|---|---|
| Sample1 | 640.50a | 269.40a | 364.20a | 156.10a |
| Sample2 | 612.60a | 629.10a | 402.60a | 345.50a |
| Sample3 | 929.90a | 642.50a | 339.10a | 322.20a |
| Sample4 | 665.10a | 537.10a | 773.10a | 403.40a |
| Sample5 | 628.60a | 156.10a | 407.50a | 598.40a |
| Sample6 | 441.30a | 345.50a | 514.90a | 391.50a |
| Average | 653.0±54 | 429.95±69 | 466.90±55 | 369.52±49 |

Fungi

| Simpson | PM | BM | PC | BC |
|---|---|---|---|---|
| sample1 | 0.9062a | 0.8429b | 0.8751a | 0.8172a |
| sample2 | 0.7315a | 0.8003b | 0.9064a | 0.8591a |
| sample3 | 0.8511a | 0.6016b | 0.7667a | 0.5569a |
| sample4 | 0.9056a | 0.5739b | 0.8282a | 0.8444a |
| sample5 | 0.9177a | 0.7372b | 0.8492a | 0.824a |
| sample6 | 0.8836a | 0.7349b | 0.8664a | 0.8216a |
| Average | 5.1957±0.02 | 4.2908±0.01 | 5.092±0.02 | 4.7232±0.04 |

| Shannon | PM | BM | PC | BC |
|---|---|---|---|---|
| sample1 | 3.557a | 2.911a | 2.902a | 2.904aa |
| sample2 | 2.16a | 2.313a | 2.91a | 2.988 |
| sample3 | 2.742a | 1.676a | 1.541a | 2.08a |
| sample4 | 3.042a | 1.798a | 2.486a | 2.393a |
| sample5 | 3.296a | 2.037a | 2.856a | 2.324a |
| sample6 | 2.813a | 2.049a | 3.087a | 2.556a |
| Average | 2.935±0.19 | 2.130±0.18 | 2.630±0.23 | 2.540±0.24 |

| Chao | PM | BM | PC | BC |
|---|---|---|---|---|
| sample1 | 388a | 385.3a | 380.2a | 291a |
| sample2 | 337.2a | 307.6a | 347.5a | 299.2a |
| sample3 | 333.1a | 331.6a | 236.6a | 272a |
| sample4 | 343.5a | 356.4a | 246a | 177.3a |
| sample5 | 296.7a | 298.1a | 395.8a | 172.6a |
| sample6 | 370.1a | 281a | 417.1a | 194.3a |
| Average | 344.7667±12 | 326.6667±15 | 337.2±31 | 234.4±24 |

Supplementary material Table 5

## 5a Significant difference between number of Dominant OTUs from 16s RNA genes

| | Rosenbergiella | Paracoccus | Core gut bacteria | Pantoea | Buttiauxella | Pseudomonas | Acinetobacter | Escherichia-Shigella |
|---|---|---|---|---|---|---|---|---|
| PM/PC | 0.0190 | 0.0200 | 0.0010 | 0.0900 | 0.0040 | 0.1500 | 0.2500 | 0.4700 |
| PM/BM | 0.0200 | 0.0004 | 0.0100 | 0.5700 | 0.0010 | 0.0040 | 0.0001 | 0.0001 |
| PC/BC | 0.0200 | 0.0040 | 0.0450 | 0.4300 | 0.0680 | 0.0050 | 0.0030 | 0.0010 |
| BC/BM | 0.0030 | 0.0040 | 0.0037 | 0.0020 | 0.1200 | 0.2600 | 0.9300 | 0.0100 |

## 5b Significant difference between number of Dominant OTUs from ITS genes

| | Cladosporium | Botrytis | unclassified__Sclerotiniaceae | unclassified_Trichosphaeriales | Rhodosporidium | Penicillium | Aspergillus | Alternaria |
|---|---|---|---|---|---|---|---|---|
| PM/PC | 0.47 | 0.18 | 0.18 | 0.03 | 1 | 0.55 | 0.04 | 0.39 |
| PM/BM | 0.11 | 0.76 | 0.55 | 1 | 0.049 | 0.03 | 0.029 | 1 |
| PC/BC | 0.82 | 1 | 1 | 0.03 | 1 | 0.07 | 0.03 | 0.55 |
| BC/BM | 0.31 | 1 | 0.27 | 1 | 0.32 | 1 | 1 | 1 |

Supplementary material Table 6

Number of gene prediction group and significant difference between corbicular pollen and hive stored bee bread

| COG (clusters of orthologous groups) | Corbicular pollen | Hive stored bee bread | P value |
|---|---|---|---|
| RNA processing and modification | 1283.5±94 | 1256±137 | 0.88 |
| Chromatin structure and dynamics | 34174±511 | 39900.5±1116 | 0.06 |
| Cell cycle control, cell division, chromosome partitioning | 853655±1694 | 847689.5±195 | 0.88 |
| Amino acid transport and metabolism | 4176329±596 | 5172730±371 | 0.03 |
| Nucleotide transport and metabolism | 1437949±507 | 1482710±525 | 0.88 |
| Carbohydrate transport and metabolism | 2582917±439 | 2927503±133 | 0.06 |
| Coenzyme transport and metabolism | 3169731±250 | 3411926±129 | 0.88 |
| Lipid transport and metabolism | 1633865±538 | 1691349±559 | 0.88 |
| Translation, ribosomal structure and biogenesis | 4489865±917 | 3834958±1120 | 0.07 |
| Transcription | 2542563±340 | 2781095±120 | 0.88 |
| Replication, recombination and repair | 4885050±564 | 4890091±1660 | 0.88 |
| Cell wall/membrane/envelope biogenesis | 4508521±352 | 4833441±1783 | 0.88 |
| Cell motility | 477751±141 | 562342.5±308 | 0.31 |
| Posttranslational modification, protein turnover, chaperones | 3672480±536 | 3705697±1256 | 0.88 |
| Inorganic ion transport and metabolism | 3639585±5921 | 4157809±1592 | 0.31 |
| Secondary metabolites biosynthesis, transport and catabolism | 937482.5±100 | 1033173±3672 | 0.3 |
| Signal transduction mechanisms | 4975524±6810 | 5595125±1990 | 0.32 |
| Intracellular trafficking, secretion, and vesicular transport | 1609635±1908 | 1467044±5408 | 0.34 |
| Defense mechanisms | 1248880±1578 | 1383306±4789 | 0.3 |

Supplementary material Table 7

Isolation and characterization of filamentous fungi associated with corbicular pollen and hive storage bee bread. (Shaded field means-presence/activity)

| Isolates | The most closely related with NCBI | Percent | Accession number of references | Length s | Acid | Antagonist with Chalkbrood | | Protease | Lipas e |
|---|---|---|---|---|---|---|---|---|---|
| | | | | | | Faster growing | inhibition growing | | |
| PM1 | *Trichoderma longibrachiatum* | 99 | KM225908.1 | 667 | | | | | |
| PM2 | *Penicillium glabrum* | 100 | MH854998.1 | 812 | | | | | |
| PM3 | *Fusarium boothii* | 100 | NR_121203.1 | 598 | | | | | |
| PM5 | *Cladosporium tenuissimum* | 100 | MH864840.1 | 699 | | | | | |
| PM6 | *Trichoderma longibrachiatum* | 98 | KM225908.1 | 667 | | | | | |
| Pm7 | *Aspergillus costaricaensis* | 100 | MH862988.1 | 598 | | | | | |
| PM9 | *Trichoderma longibrachiatum* | 99 | KM225908.1 | 667 | | | | | |
| PM10 | *Penicillium glabrum* | 99 | MH854998.1 | 812 | | | | | |
| PC1 | *Trichoderma longibrachiatum* | 100 | KM225908.1 | 668 | | | | | |
| PC2 | *Penicillium sumatraense* | 99 | NR_119812.1 | 543 | | | | | |
| PC3 | *Penicillium citreosulfuratum* | 100 | NR_153252.1 | 679 | | | | | |
| PC4 | *Pestalotiopsis kenyana* | 100 | NR_147549.1 | 597 | | | | | |
| PC5 | *Penicillium consobrinum* | 99 | MG490874.1 | 809 | | | | | |
| BM2 | *Aspergillus foetidus* | 100 | MH854949.1 | 601 | | | | | |
| BM3 | *Mucor fragilis* | 98 | FN650655.1 | 660 | | | | | |
| BM4 | *Pestalotiopsis kenyana* | 99 | NR_147549.1 | 597 | | | | | |
| BM5 | *Alternaria angustiovoidea* | 100 | MH861939.1 | 568 | | | | | |
| BM6 | *Penicillium thomii* | 100 | NR_077159.1 | 602 | | | | | |
| BM7 | *Epicoccum thailandicum* | 99 | NR_152926.1 | 513 | | | | | |
| Isolates | The most closely related with NCBI | Percent | | Length s | Acid | Antagonist with Chalkbrood | | Protease | Lipas e |

| | | | Accession number of references | | | Faster growing | inhibition growing | | |
|---|---|---|---|---|---|---|---|---|---|
| BM8 | *Paraconiothyrium brasiliense* | 100 | JQ936270.1 | 558 | | | | | |
| BM9 | *Cladosporium parahalotolerans* | 99 | MF473164.1 | 837 | | | | | |
| BM10 | *Trichoderma longibrachiatum* | 99 | KM225908.1 | 667 | | | | | |
| BM11 | *Trichoderma harzianum* | 99 | MF780869.1 | 660 | | | | | |
| BM12 | *Trichoderma longibrachiatum* | 99 | KM225892.1 | 667 | | | | | |
| BM13 | *Aspergillus foetidus* | 99 | MH854949.1 | 601 | | | | | |
| BM14 | *Fusarium oxysporum* | 100 | MH290453.1 | 572 | | | | | |
| BM15 | *Alternaria angustiovoidea* | 100 | MH861939.1 | 568 | | | | | |
| BM16 | *Mucor fragilis* | 99 | MF467898.1 | 668 | | | | | |
| BC2 | *Cladosporium ramotenellum* | 100 | MG548565.1 | 579 | | | | | |
| BC3 | *Mucor fragilis* | 100 | GU566275.1 | 675 | | | | | |
| BC4 | *Talaromyces scorteus* | 99 | MH857965.1 | 605 | | | | | |
| BC5 | *Mucor fragilis* | 100 | GU566275.1 | 675 | | | | | |
| BC6 | *Talaromyces dendriticus* | 100 | MH861305.1 | 604 | | | | | |
| GZ | *Ascosphaera apis* | 99 | MH862580.1 | 647 | | | | | |
| SX | *Ascosphaera apis* | 99 | MH862580.1 | 648 | | | | | |

Supplementary material Table8

Isolation and characterization of bacteria associated with corbicular pollen and hive storage bee bread (Shaded field means-presence/activity)

| Isolate | The most closely related with NCBI | Percent | Accession number of references | Length | Acid | Antagonist with Chalkbrood | | Protease | Lipase |
|---|---|---|---|---|---|---|---|---|---|
| | | | | | | Faster growing | inhibition growing | | |
| PM1 | *Buttiauxella gaviniae* | 98 | NR_025330.1 | 1495 | | | | | |
| PM2 | *Pantoea ananatis* | 99 | NR_026045.1 | 1455 | | | | | |
| PC2 | *Pantoea ananatis* | 99 | NR_026045.1 | 1455 | | | | | |
| PC1 | *Buttiauxella gaviniae* | 98 | NR_025330.1 | 1495 | | | | | |
| BC2 | *Acinetobacter soli* | 99 | NR_044454.1 | 1422 | | | | | |

Supplementary material table 9

Positive antagonistic of filamentous fungal supernatant to bacterial isolates (Shaded field means-presence/activity)

<table>
<tr><td></td><td>Bacteria</td><td>PM1</td><td>PM2</td><td>PC1</td><td>PC2</td><td>BC2</td></tr>
<tr><td>Fungi</td><td>taxa</td><td>Buttiauxella gaviniae</td><td>Pantoea ananatis</td><td>Buttiauxella gviniae</td><td>Pantoea sp.</td><td>Acinetobacter soli</td></tr>
<tr><td>PM1</td><td>Trichoderma longibrachiatum</td><td></td><td></td><td style="background:#c0c0c0"></td><td style="background:#c0c0c0"></td><td style="background:#c0c0c0"></td></tr>
<tr><td>PM7</td><td>Aspergillus niger</td><td style="background:#c0c0c0"></td><td style="background:#c0c0c0"></td><td style="background:#c0c0c0"></td><td style="background:#c0c0c0"></td><td style="background:#c0c0c0"></td></tr>
<tr><td>PM9</td><td>Trichoderma longibrachiatum</td><td></td><td></td><td></td><td></td><td style="background:#c0c0c0"></td></tr>
<tr><td>PC1</td><td>Trichoderma longibrachiatum</td><td style="background:#c0c0c0"></td><td style="background:#c0c0c0"></td><td></td><td></td><td style="background:#c0c0c0"></td></tr>
<tr><td>PC2</td><td>Penicillium sp.</td><td></td><td></td><td></td><td></td><td style="background:#c0c0c0"></td></tr>
<tr><td>PC3</td><td>Penicillium citreosulfuratum</td><td></td><td></td><td></td><td></td><td style="background:#c0c0c0"></td></tr>
<tr><td>PC5</td><td>Penicillium consobrinum</td><td style="background:#c0c0c0"></td><td style="background:#c0c0c0"></td><td style="background:#c0c0c0"></td><td style="background:#c0c0c0"></td><td style="background:#c0c0c0"></td></tr>
<tr><td>BM2</td><td>Aspergillus niger</td><td style="background:#c0c0c0"></td><td style="background:#c0c0c0"></td><td style="background:#c0c0c0"></td><td style="background:#c0c0c0"></td><td></td></tr>
<tr><td>BM9</td><td>Cladosporium parahalotolerans</td><td style="background:#c0c0c0"></td><td></td><td style="background:#c0c0c0"></td><td></td><td style="background:#c0c0c0"></td></tr>
</table>

Supplementary material figure1

The proportion graph of total bacterial phylum (1a) and genera (1b)

1a)

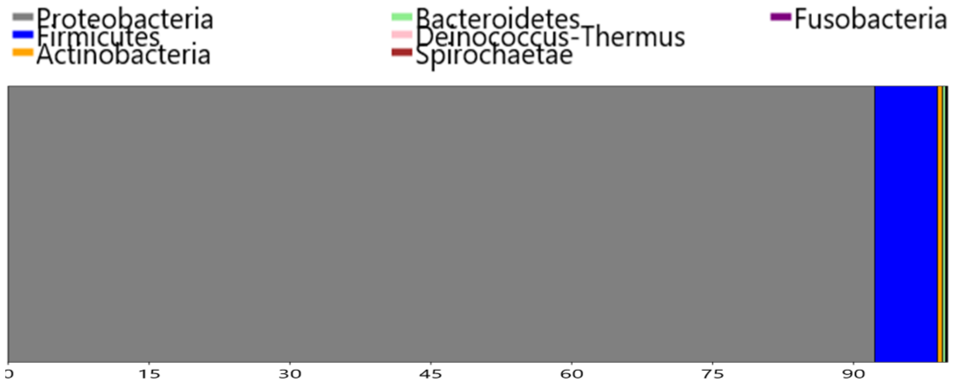


1b)

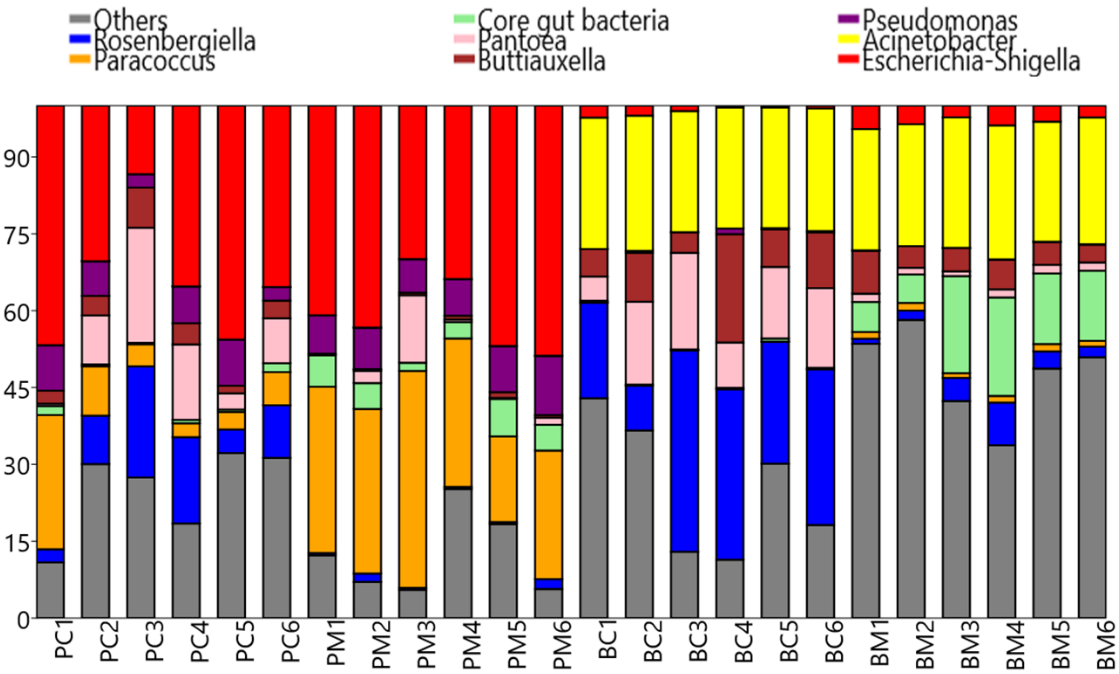

Supplementary material figure 2

The proportion of total fungal phylum (2a) and genera (2b)

2a)

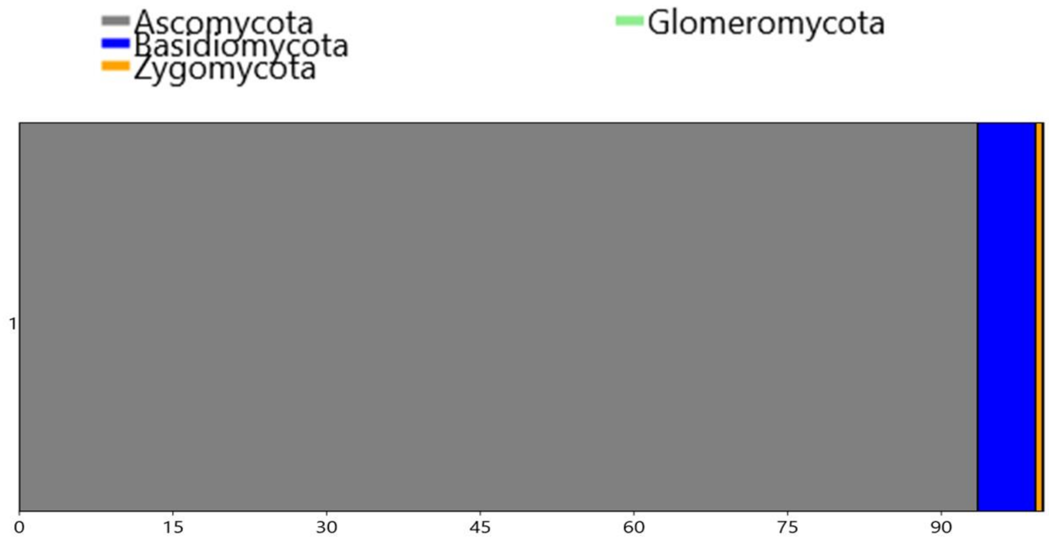


2b)

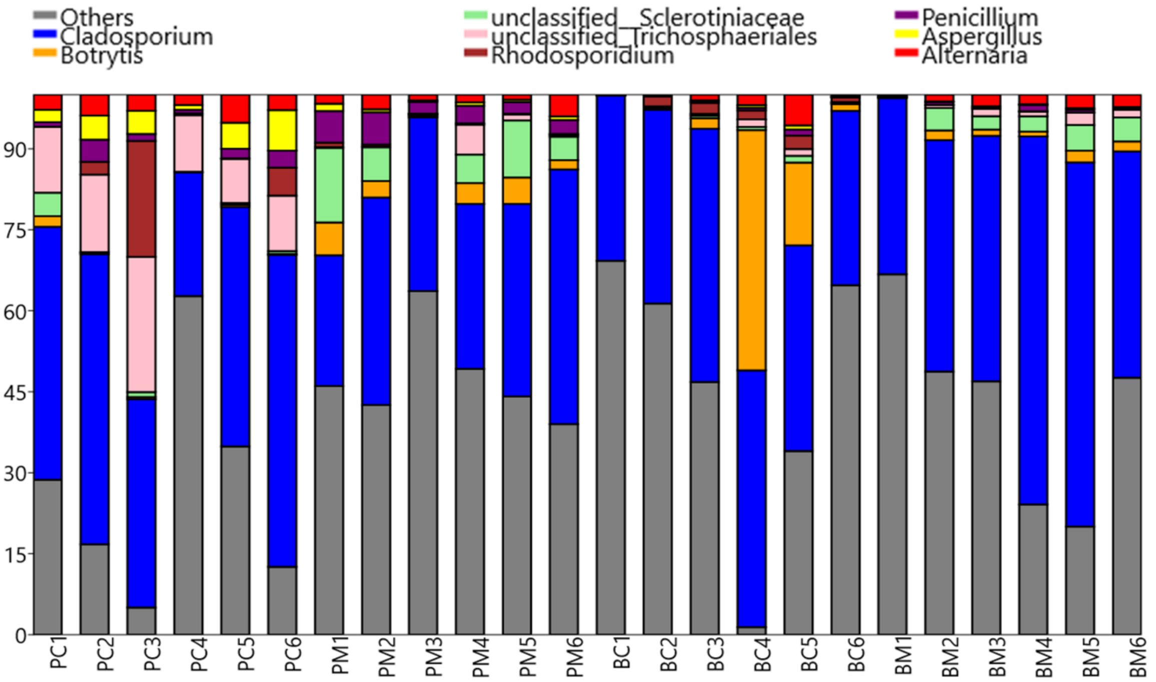

Supplementary material figure 3

The bacterial (3a) and fungal (3b) taxon impacted on communities associated with corbicular pollen (P) and hive storage bee bread (B) via LDA scores

3a)

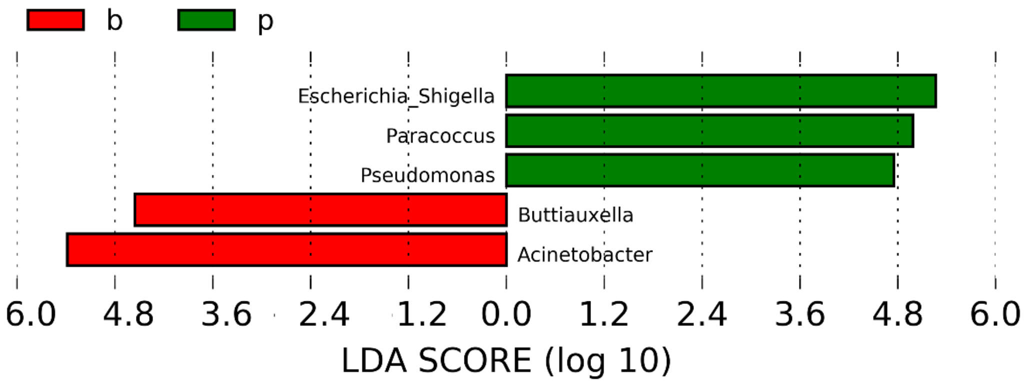


3b)

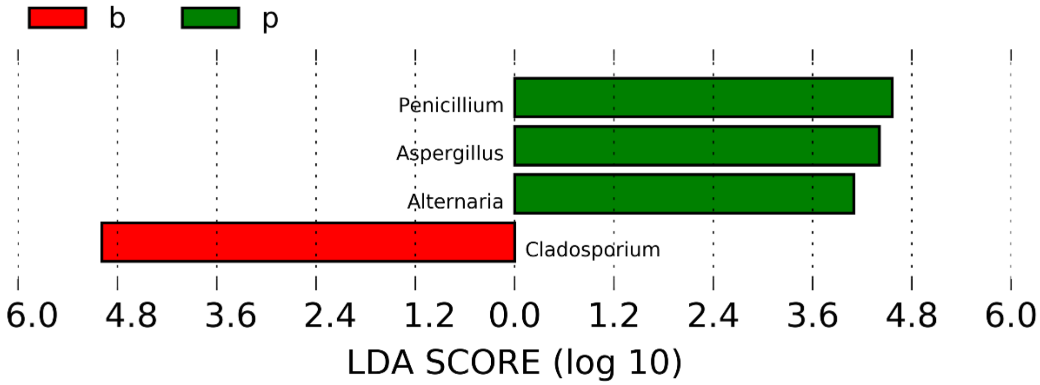

Supplementary material figure4

Graph of total COG functional genes from 16s rRNA data base on Greengene database (4a) and their affect on group of investigation by LDA score (4b)

4a)

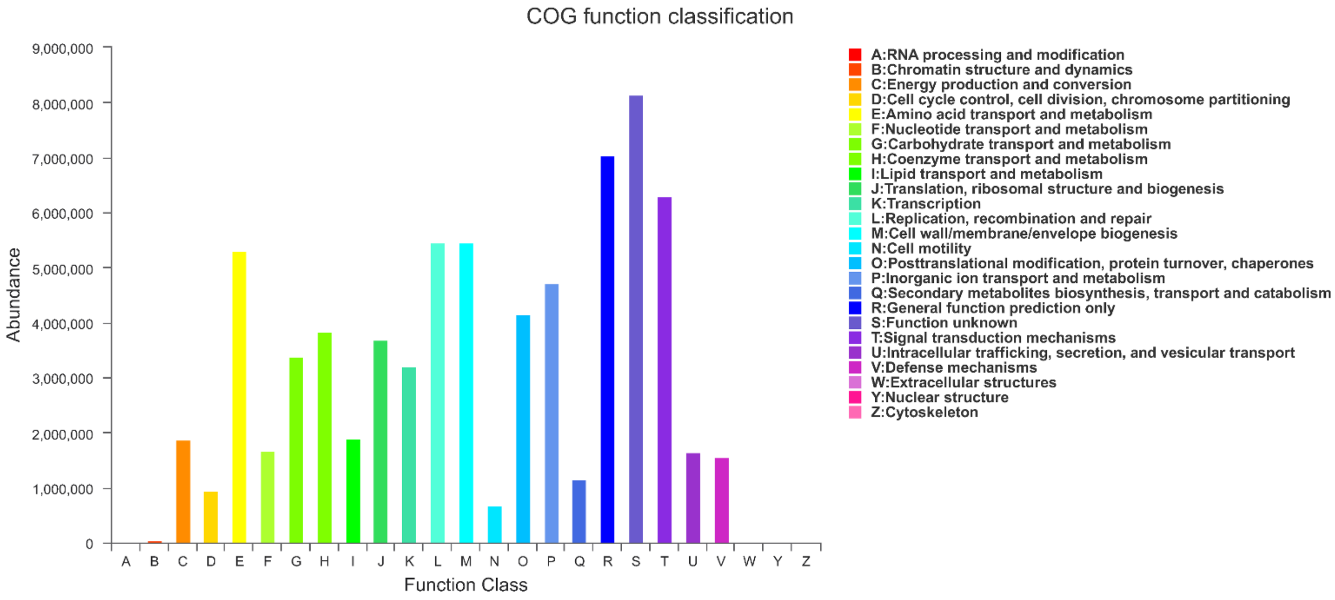


4b)

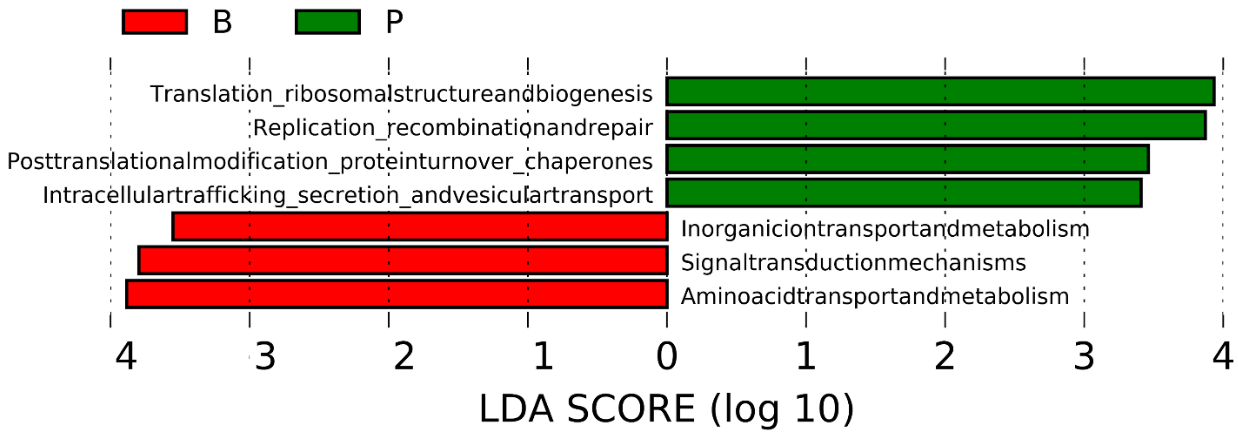

Supplementary material figure 5

Phylogenetics tree base on neighbor joining tree of fungal (5a,b ) and bacterial isolates (5c) from all samples

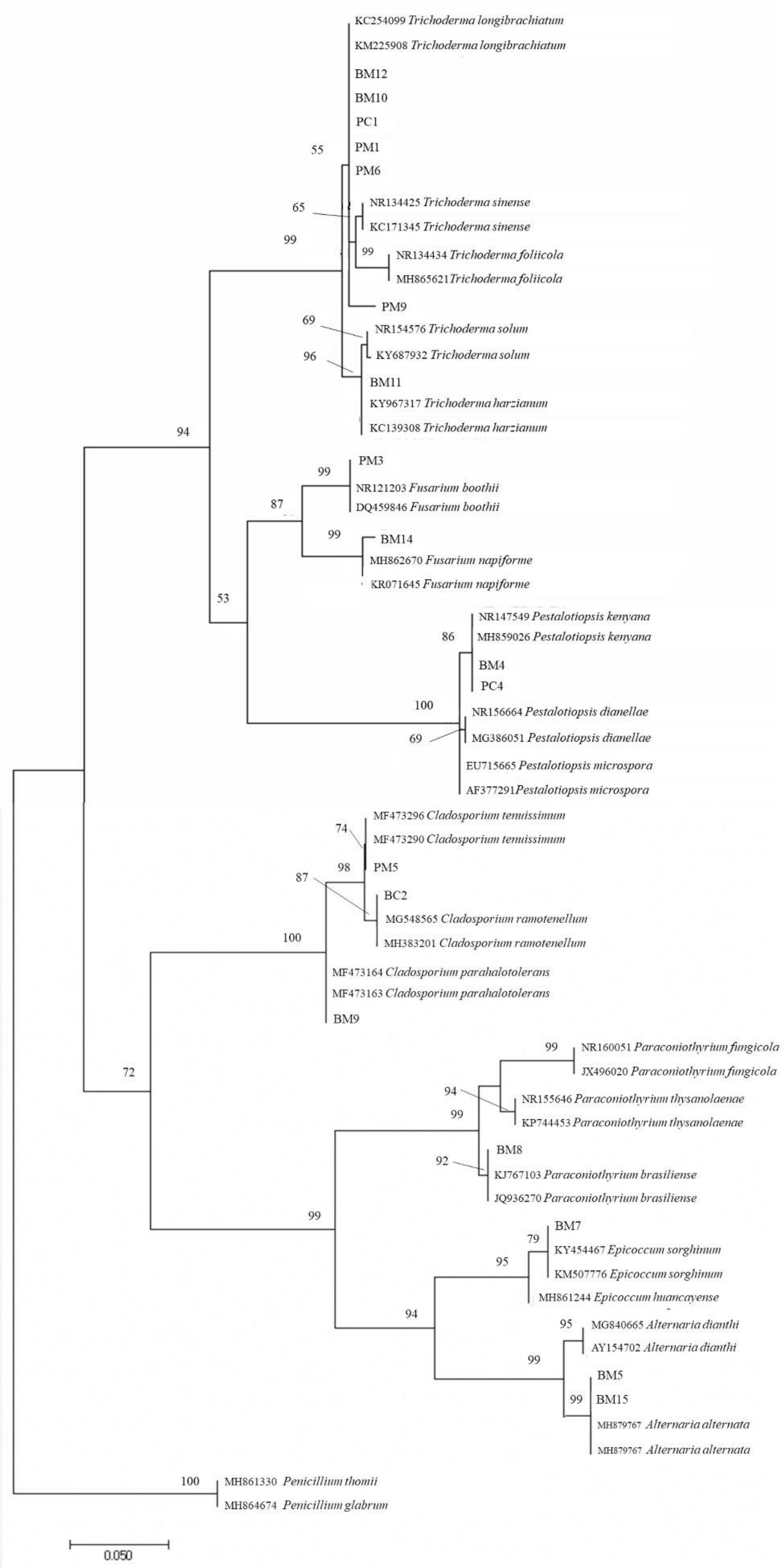
KC254099 Trichoderma longibrachiatum
KM225908 Trichoderma longibrachiatum
BM12
BM10
PC1
PM1
PM6
NR134425 Trichoderma sinense
KC171345 Trichoderma sinense
NR134434 Trichoderma foliicola
MH865621 Trichoderma foliicola
PM9
NR154576 Trichoderma solum
KY687932 Trichoderma solum
BM11
KY967317 Trichoderma harzianum
KC139308 Trichoderma harzianum
PM3
NR121203 Fusarium boothii
DQ459846 Fusarium boothii
BM14
MH862670 Fusarium napiforme
KR071645 Fusarium napiforme
NR147549 Pestalotiopsis kenyana
MH859026 Pestalotiopsis kenyana
BM4
PC4
NR156664 Pestalotiopsis dianellae
MG386051 Pestalotiopsis dianellae
EU715665 Pestalotiopsis microspora
AF377291 Pestalotiopsis microspora
MF473296 Cladosporium tenuissimum
MF473290 Cladosporium tenuissimum
PM5
BC2
MG548565 Cladosporium ramotenellum
MH383201 Cladosporium ramotenellum
MF473164 Cladosporium parahalotolerans
MF473163 Cladosporium parahalotolerans
BM9
NR160051 Paraconiothyrium fungicola
JX496020 Paraconiothyrium fungicola
NR155646 Paraconiothyrium thysanolaenae
KP744453 Paraconiothyrium thysanolaenae
BM8
KJ767103 Paraconiothyrium brasiliense
JQ936270 Paraconiothyrium brasiliense
BM7
KY454467 Epicoccum sorghinum
KM507776 Epicoccum sorghinum
MH861244 Epicoccum huancayense
MG840665 Alternaria dianthi
AY154702 Alternaria dianthi
BM5
BM15
MH879767 Alternaria alternata
MH879767 Alternaria alternata
MH861330 Penicillium thomii
MH864674 Penicillium glabrum
55
65
99
99
69
96
94
99
87
99
53
86
100
69
74
98
87
100
72
99
94
99
92
99
79
95
94
95
99
99
100
0.050

5a)

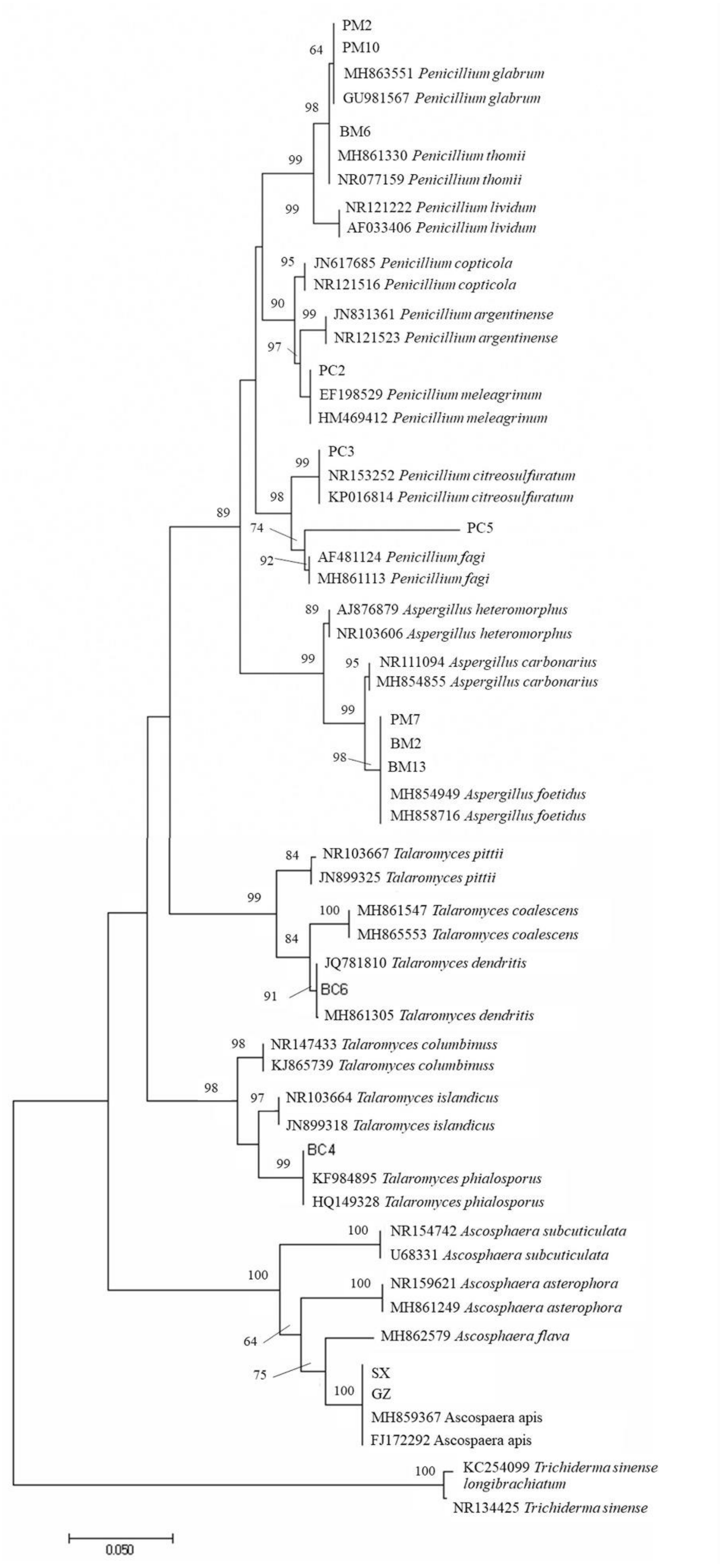
PM2
PM10
MH863551 Penicillium glabrum
GU981567 Penicillium glabrum
BM6
MH861330 Penicillium thomii
NR077159 Penicillium thomii
NR121222 Penicillium lividum
AF033406 Penicillium lividum
JN617685 Penicillium copticola
NR121516 Penicillium copticola
JN831361 Penicillium argentinense
NR121523 Penicillium argentinense
PC2
EF198529 Penicillium meleagrinum
HM469412 Penicillium meleagrinum
PC3
NR153252 Penicillium citreosulfuratum
KP016814 Penicillium citreosulfuratum
PC5
AF481124 Penicillium fagi
MH861113 Penicillium fagi
AJ876879 Aspergillus heteromorphus
NR103606 Aspergillus heteromorphus
NR111094 Aspergillus carbonarius
MH854855 Aspergillus carbonarius
PM7
BM2
BM13
MH854949 Aspergillus foetidus
MH858716 Aspergillus foetidus
NR103667 Talaromyces pittii
JN899325 Talaromyces pittii
MH861547 Talaromyces coalescens
MH865553 Talaromyces coalescens
JQ781810 Talaromyces dendritis
BC6
MH861305 Talaromyces dendritis
NR147433 Talaromyces columbinuss
KJ865739 Talaromyces columbinuss
NR103664 Talaromyces islandicus
JN899318 Talaromyces islandicus
BC4
KF984895 Talaromyces phialosporus
HQ149328 Talaromyces phialosporus
NR154742 Ascosphaera subcuticulata
U68331 Ascosphaera subcuticulata
NR159621 Ascosphaera asterophora
MH861249 Ascosphaera asterophora
MH862579 Ascosphaera flava
SX
GZ
MH859367 Ascospaera apis
FJ172292 Ascospaera apis
KC254099 Trichiderma sinense longibrachiatum
NR134425 Trichiderma sinense
0.050

5b)

5c)

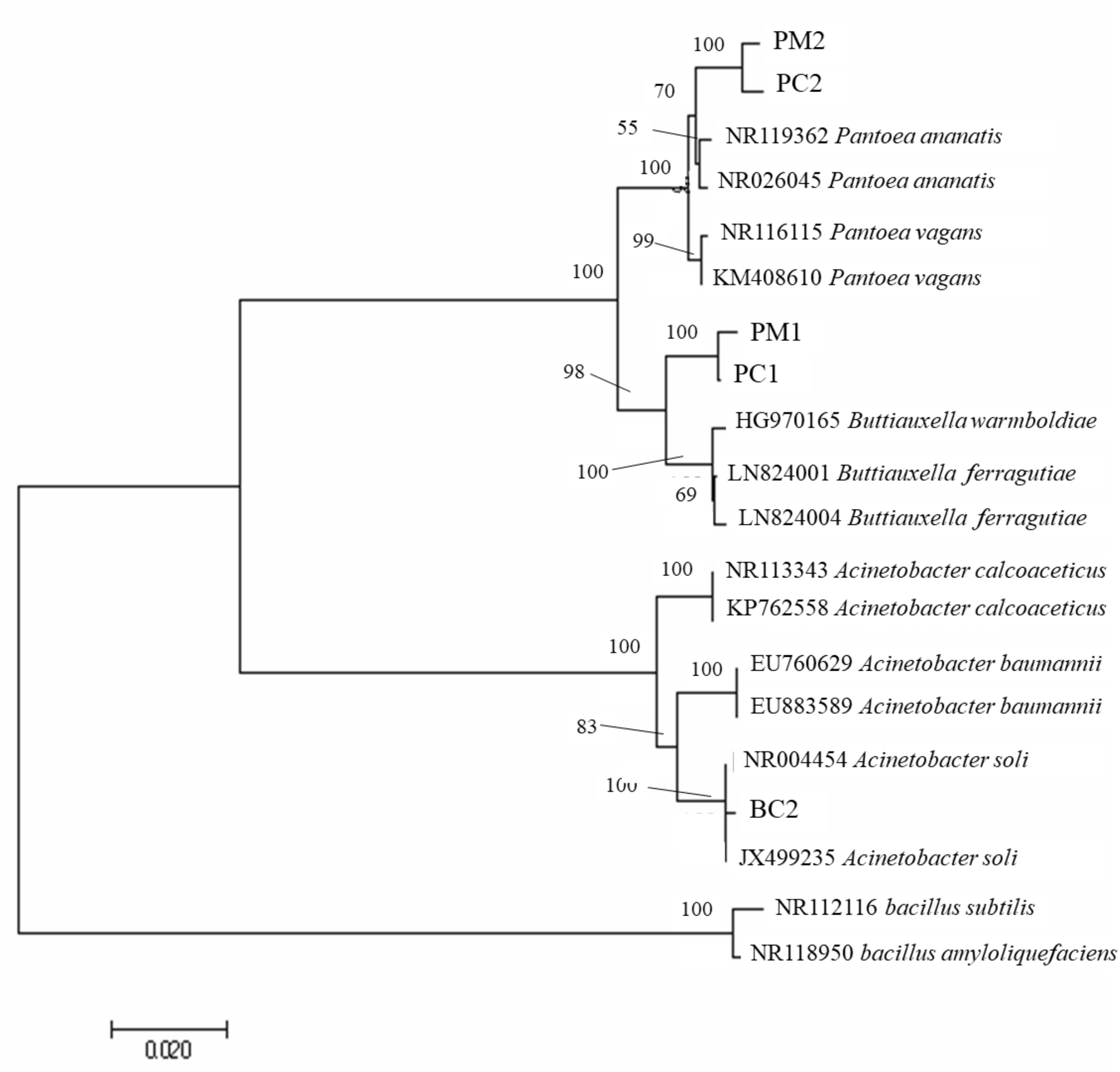

100
PM2
PC2
70
55
NR119362 *Pantoea ananatis*
100
NR026045 *Pantoea ananatis*
NR116115 *Pantoea vagans*
99
100
KM408610 *Pantoea vagans*
100
PM1
PC1
98
HG970165 *Buttiauxella warmboldiae*
100
LN824001 *Buttiauxella ferragutiae*
69
LN824004 *Buttiauxella ferragutiae*
100
NR113343 *Acinetobacter calcoaceticus*
KP762558 *Acinetobacter calcoaceticus*
100
100
EU760629 *Acinetobacter baumannii*
EU883589 *Acinetobacter baumannii*
83
NR004454 *Acinetobacter soli*
100
BC2
JX499235 *Acinetobacter soli*
100
NR112116 *bacillus subtilis*
NR118950 *bacillus amyloliquefaciens*
0.020

Supplementary material figure 6

The example of bioactivity screening from fungal isolates

6a) The fungal isolate release organic acid change indicator from green to yellow

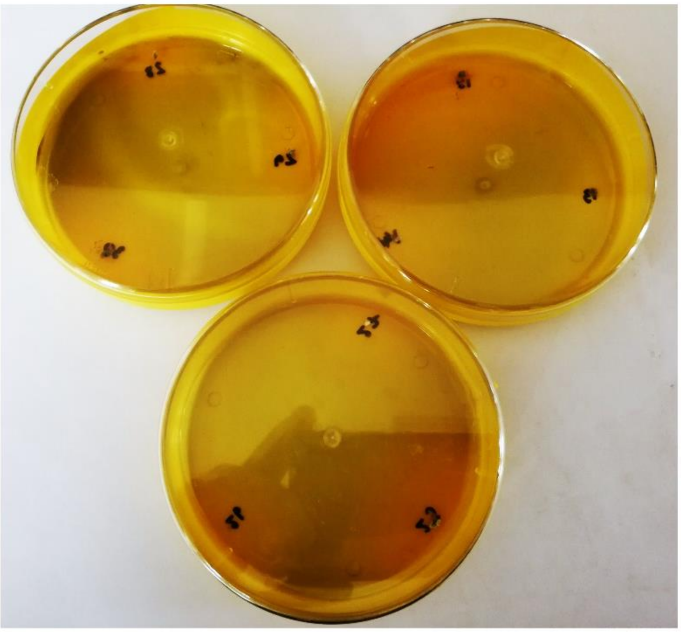

6b) Faster growing of fungal isolate monopolizes chalkbrood disease, this figure from isolate PM6

**Chalkbrood**

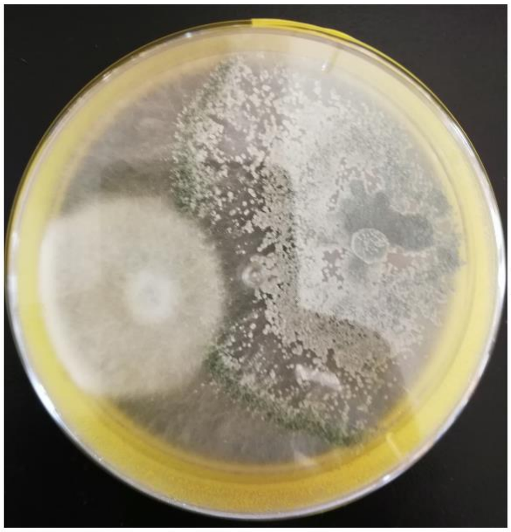

**Isolate PM6**

6c) Antagonistic inhibition of fungal isolate with chalkbrood disease, this figure from isolate BM1

**Chalkbrood**

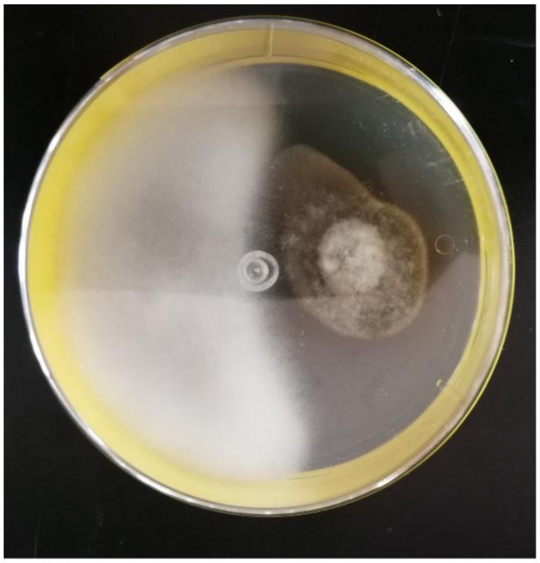

**Isolate BM1**

6d) protease enzyme screening of fungal isolate PM5

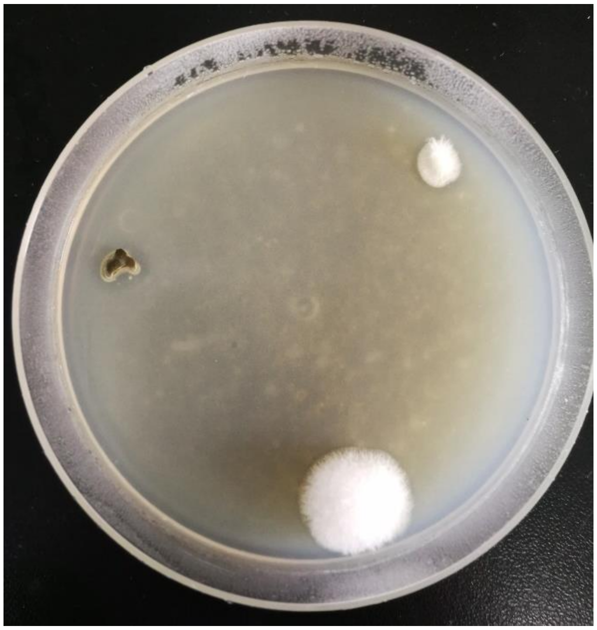

6e) lipase enzyme screening of fungal isolate BC2 (45)

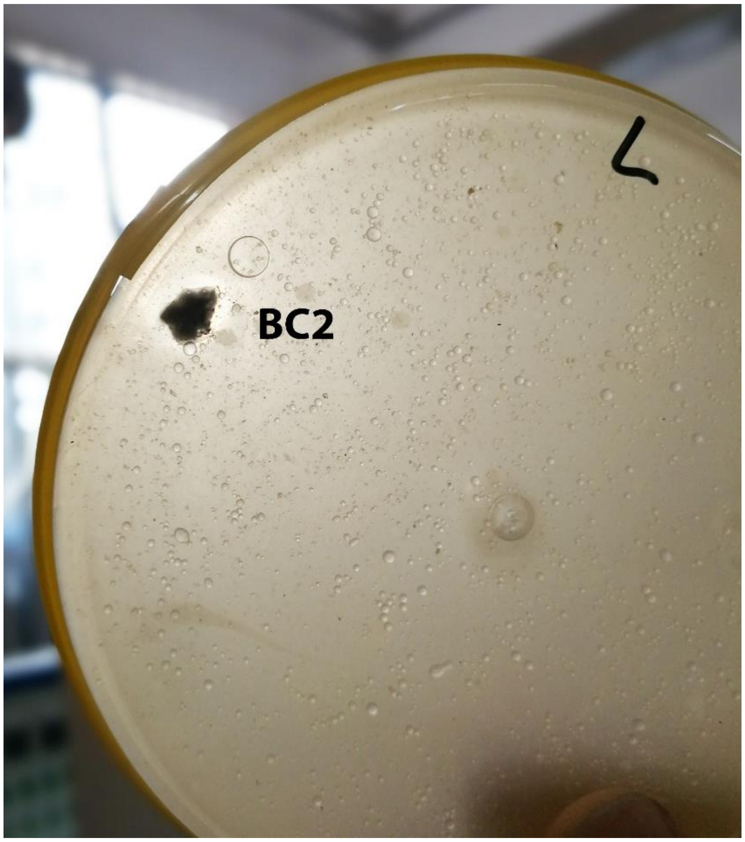

6f) fungal supernatant PM1(24) and PM7 (32) inhibit bacterial isolates PC1,1% ampicillin is positive control and water is negative control

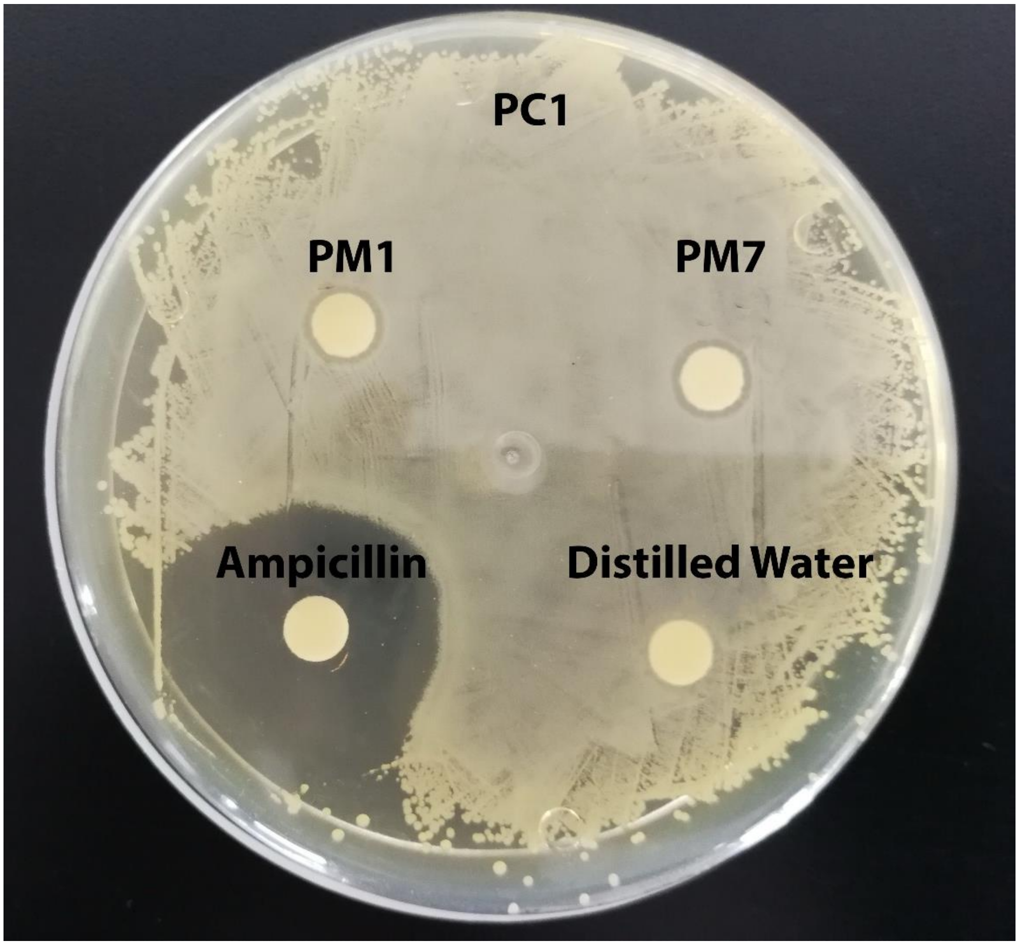

Supplement: Supplementary file 1 [file microorganisms-08-00264-s001.pdf]
